# Supplementary material for: The Use of Cabozantinib in Advanced Hepatocellular Carcinoma in Hong Kong—A Territory-Wide Cohort Study
Source: Cancers (Basel). 2021 Apr 21;13(9):2002. doi: 10.3390/cancers13092002 (PMC8122581; doi:10.3390/cancers13092002)
Supplement: Supplementary file 1 [file cancers-13-02002-s001.zip › cancers-1175034-supplementary/cancers-1175034_supplementary/Cancers Supp Table 1.pdf]

Table S1. Detailed prior systemic treatments

Previous therapies, *n* (%)	All patients	Single Agent (*n* = 27)	Combination (*n* = 15)
Curative Resection	21 (50%)	9 (33.3%)	12 (80%)
Radiotherapy	10 (23.8%)	6 (22.2%)	4 (26.7%)
Transarterial chemoembolization (TACE)	20 (47.6%)	13 (48.1%)	7 (46.7%)
Liver Transplant	1 (2.4%)	1 (3.7%)	0
Any TKI	40 (95.2%)	26 (96.3%)	14 (93.3%)
Lenvatinib	28 (66.7%)	15 (55.6%)	13 (86.7%)
Sorafenib	17 (40.5%)	14 (51.9%)	3 (20%)
Regorafenib	3 (7.1%)	2 (7.4%)	1 (6.7%)
Axitinib	2 (4.8%)	2 (7.4%)	0
Any ICI	31 (73.8%)	16 (59.3%)	15 (100%)
Anti-PD1	19 (45.2%)	12 (44.4%)	7 (46.7%)
Anti-PD1 & Anti-CTLA-4	12 (28.6%)	5 (18.5%)	7 (46.7%)
VEGF inhibitors	8 (19.0%)	1 (3.7%)	7 (46.7%)
Chemotherapy	7 (16.7%)	3 (11.1%)	4 (26.7%)
mTOR inhibitor	7 (16.7%)	2 (7.4%)	5 (33.3%)
Blue-554	1 (2.4%)	1 (3.7%)	0
NIS793+PDR001	1 (2.4%)	1 (3.7%)	0
