# Supplementary material for: The Use of Cabozantinib in Advanced Hepatocellular Carcinoma in Hong Kong—A Territory-Wide Cohort Study
Source: Cancers (Basel). 2021 Apr 21;13(9):2002. doi: 10.3390/cancers13092002 (PMC8122581; doi:10.3390/cancers13092002)
Supplement: Supplementary file 1 [file cancers-13-02002-s001.zip › cancers-1175034-supplementary/cancers-1175034_supplementary/Cancers Supp Table 2.pdf]

Supplementary Table 2. Cabozantinib combination details (*n* = 15)

n (%)		
Any ICI		15 (100%)
ICI used	Anti-PD-1 only	4 (26.7%)
	Anti-PD-1 + Anti-CTLA-4	11 (73.3%)
Others		2 (13.3%)
